# Renal pelvis mucosal artery hemorrhage after percutaneous nephrolithotomy: a rare case report and literature review

**DOI:** 10.1186/s12894-022-01049-w

**Published:** 2022-07-11

**Authors:** Lv Wen Zhang, Xiang Fei, Yan Song

**Affiliations:** Department of Urology, Shengjing Hospital of China Medical University, Shenyang, 110000 China

**Keywords:** Percutaneous nephrolithotomy, Bleeding, Renal pelvis mucosal artery, Selective angioembolization

## Abstract

**Background:**

Following a percutaneous nephrolithotomy (PCNL) procedure, the most common complications are considered to be intraoperative and postoperative bleeding. Many patients with postoperative bleeding can be treated conservatively, causing the perirenal hematoma to resolve spontaneously. The major causes of severe postoperative bleeding are pseudoaneurysms, arteriovenous fistula, and segmental arterial injury. Typically, the first choice of treatment to manage severe bleeding complications is selective angioembolization (SAE) because of the very high success rate associated with this procedure.

**Case presentation:**

This clinical case involves a 56-year-old man who underwent dual-channel PCNL treatment after diagnosing a left kidney staghorn stone and urinary tract infection. The operation was successful, with no apparent signs of bleeding. Tests revealed continued decreasing hemoglobin levels following the procedure. After the conservative treatment failed, renal angiography was performed immediately, indicating renal pelvis mucosal artery hemorrhage. In the three hours post-surgery, the SAE still failed to prevent bleeding. Further discussions led to formulating a new surgical plan using a nephroscope to enter the initial channel where hemostasis began. The hemostasis origin was found precisely in the mucosal artery next to the channel during the operation and was successfully controlled.

**Conclusions:**

This case reveals there is poor communication and inadequate discussions about the potential failures of an SAE procedure. Swift clinical decision-making is imperative when dealing with high-level renal trauma to prevent delays in surgery that can threaten the safety of patients.

**Supplementary Information:**

The online version contains supplementary material available at 10.1186/s12894-022-01049-w.

## Background

Technological advancements in surgery have developed minimally invasive endoscopic procedures like PCNL, which has replaced traditional open surgery as the first choice for treating large kidney stones [1]. Although PCNL is a technique that offers a significantly high stone removal rate and low recurrence rate as a primary treatment option, hemorrhage is still the most common complication of PCNL. Typical hemorrhages diagnosed by a renal angiography procedure are pseudoaneurysm, arteriovenous fistula, and staged arterial injury; severe bleeding requires superselective vascular embolization [2, 3]. The latter has a very high success rate, but 0.2% of patients with embolization failure are still at risk of nephrectomy [4]. What follows is a report of a case of PCNL bleeding after the failure of superselective vessel embolization and successful hemostasis after nephroscopy procedure.

## Case presentation

**Fig. 1:**
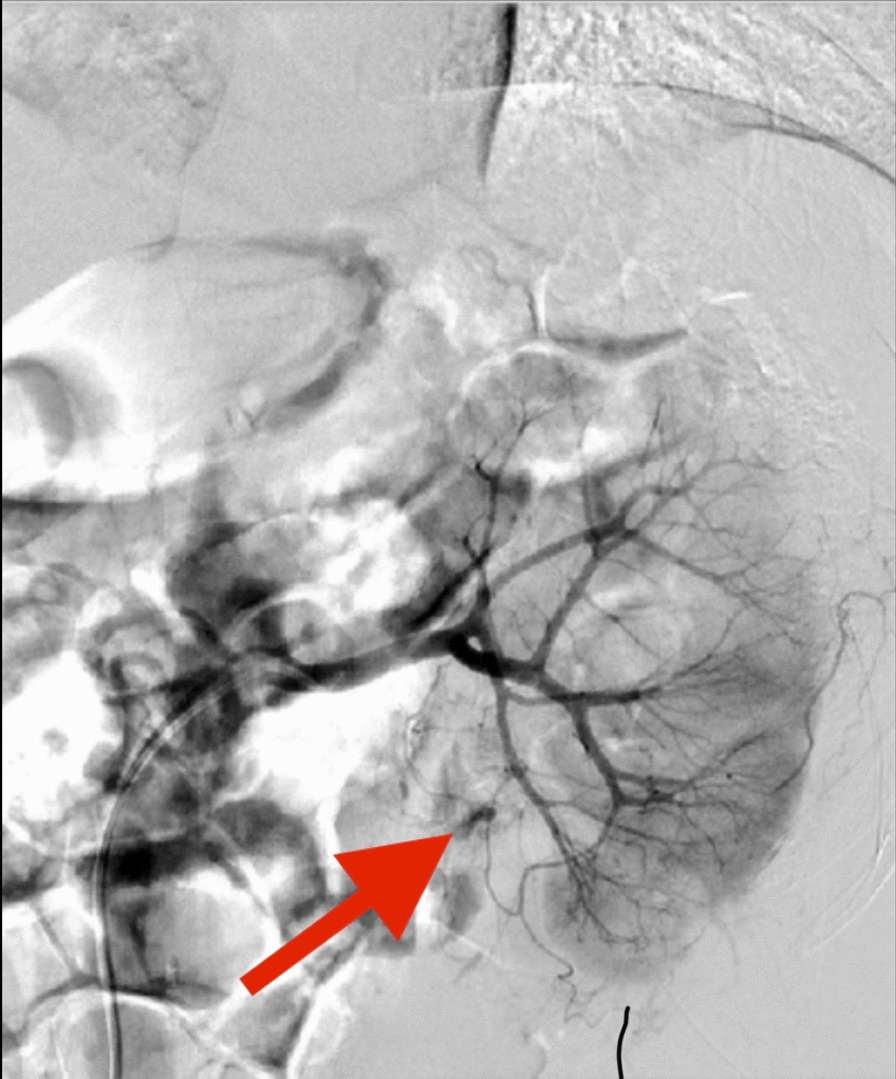
The arrow indicates rupture and bleeding of the mucosal artery of the renal pelvis

The patient was a 56-year-old male. A computerized tomography (CT) scan revealed a staghorn stone in the left kidney. The puncture point was selected under ultrasound guidance, and a vertical incision was made through the skin into the renal calyces of the left kidney. Using a 24-Fr fascial dilator to establish a clear passage, it is determined that the left renal lower calyx stone cannot be broken. To reevaluate the procedure, another puncture point is created, with a vertical skin incision into the left lower renal calyx group. The passage was dilated to 18-Fr, resulting in a successful operation process by completely removing the stone; F20 and F16 drainage tubes were retained. Post-surgery, the color of the fluid from drainage was dark red, and the hemoglobin decreased by 10 g/d. After the conservative treatment failed, the CT scan revealed renal pelvis mucosal artery hemorrhage (Fig. 1). A selective angioembolization (SAE) was performed immediately, but the operation was considered unsuccessful after 3 h of intervention therapy failed to control bleeding. There were several reasons for the failure of SAE: the blood vessels were too thin; the opening was at an angle opposite to the renal artery trunk; the renal artery trunk was thick, therefore unable to support it to enter its opening, and was too deep into the distal end, and the opening may have been at a complicated angle. Considering the patient's condition may deteriorate, the decision was made to immediately prepare for emergency hemostasis surgery. This necessity was explained to the patient’s family, along with the potential consequences of nephrectomy if hemostasis failed to achieve further hemostasis. After the patient was anesthetized, the kidney was entered along the original channel. The nephroscope cleared the clot in the renal pelvis, and the mucosal bleeding point at the junction of the renal pelvis and ureter was identified. Electrocoagulation treatment was used to successfully control bleeding after surgery. Careful inspection of the fluid in each cup revealed no bleeding or large stones remained (Additional file 1: Video 1). The hemoglobin value of the patient was monitored gradually after the operation, and there was no recurring bleeding after the patient was discharged from the hospital.

## Discussion and conclusions

PCNL is an effective method for treating large and complex kidney stones. Postoperative bleeding is one of the most common clinical complications following PCNL surgery. Severe bleeding complications can precipitate organ damage and even endanger the life of the patient [5, 6]. Most postoperative bleeding can be controlled by conservative treatment, and the frequency to control severe bleeding complications that require superselective vascular embolization is around 0.8% [7].

In this case, no apparent signs of bleeding were found during the operation. After the fistula was opened, the drainage fluid was discovered to be deeper in color, and the hemoglobin continued to decrease. An enhanced CT scan revealed a bleeding renal pelvis mucosal artery, but the failure of SAE required the surgeon to quickly decide on the patient's subsequent treatment. Consequently, the doctor decided to prevent further blood loss using nephroscopy procedure based on the analysis of 2 surgical videos. Blood loss was successfully controlled upon finding the exact bleeding point, and further damage to kidney function was prevented.

SAE has become the treatment of choice for severe, continuous, or intermittent bleeding after PCNL. A pseudoaneurysm, arteriovenous fistula, and segmental arterial injury are the most common effects of renal angiography [3]. Although SAE has a very high surgical success rate, some patients still experience repeated embolization failures. Those patients with hemodynamic problems cannot tolerate the angiography technique. Consequently, urgent additional treatment is required to control the development of the condition. In his analysis of patients with acute pulmonary embolism, Alireza [8] concluded that massive postoperative bleeding caused by acute embolism or other causes of PCNL must be treated with emergency partial nephrectomy or nephrectomy surgery. The principles of this type of surgery should be similar to a laparotomy surgical procedure used for patients with severe kidney damage for renal repair. Previously, there have been no reported cases of severe renal pelvis mucosal arterial bleeding leading to shock. Moreover, the successful management of severe bleeding complications after percutaneous nephroscopy also shows that nephrectomy is not the only cause of SAE failure.

Finding the exact bleeding point using a nephroscope for accurate hemostasis may also achieve unexpected results. According to previous studies many risk factors can predict the occurrence of bleeding after a PCNL procedure: multiple puncture sites; the type of kidney stones detected; high body mass index; prolonged operative time; history of ipsilateral renal calculi, and urinary tract infection are some of the causes that instigate severe bleeding after PCNL [9, 10]. In this report, the patient was considered for ipsilateral renal stone surgery while having a urinary tract infection. The risk of severe bleeding was significantly increased due to the complexity of dual-channel PCNL during the operation. This outcome also demonstrates that clinicians should be required to closely observe the changes of the patient's condition and assess the risk of postoperative complications.

For patients with severe bleeding after percutaneous nephrolithotomy and for those who experienced SAE failure, it is necessary to analyze the cause of failure and the patient’s condition immediately to ensure the integrity of the organs as much as possible.

## Supplementary Information

**Additional file 1**. Video. 1 Control the bleeding under the nephroscope.

## Data Availability

All data generated or analysed during this study are included in this published article.
